# GLP-1 Receptor Agonist Use and Survival Among Patients With Type 2 Diabetes and Brain Metastases

**DOI:** 10.1001/jamanetworkopen.2026.1311

**Published:** 2026-03-11

**Authors:** Kuan-Yu Chi, Yu Chang, Junmin Song, Chien-Min Chen, Pang-Shuo Perng, Hong-Min Lin

**Affiliations:** 1 Department of Internal Medicine, Jacobi Medical Center, Albert Einstein College of Medicine, Bronx, New York; 2 Department of Surgery, National Cheng Kung University Hospital, College of Medicine, National Cheng Kung University, Tainan, Taiwan; 3 Division of Neurosurgery, Department of Surgery, Changhua Christian Hospital, Changhua, Taiwan; 4 Department of Biomedical Sciences, National Chung Cheng University, Chiayi, Taiwan; 5 Department of Family Medicine, Chi-Mei Medical Center, Tainan, Taiwan

## Abstract

This cohort study assesses the association between glucagon-like peptide–1 receptor agonist (GLP-1 RA) use and survival in patients with type 2 diabetes and brain metastases.

## Introduction

Brain metastases (BM) are associated with substantial morbidity and mortality in patients with cancer.^1^ Comorbid type 2 diabetes (T2D) may worsen outcomes through metabolic and inflammatory pathways.^2^ Glucagon-like peptide–1 receptor agonists (GLP-1 RAs) have transformed beyond antiglycemic control, with emerging preclinical evidence suggesting potential neuroprotective and anti-inflammatory effects.^3^ However, clinical evidence evaluating their association with BM remains limited. Thus, we aimed to assess the association between GLP-1 RA use and survival in this population.

## Methods

This retrospective cohort study used the TriNetX Global Network, a federated database comprising 151 health care organizations around the world.^4^ (eAppendix 1 in Supplement 1) This research was approved by the National Cheng Kung University Hospital institutional review board. This report was prepared in accordance with the STROBE reporting guidelines for cohort studies, where applicable.

Adult patients (aged ≥18 years) with cancer with BM and T2D between January 1, 2018, and January 1, 2024, were identified. The exposed cohort included patients prescribed GLP-1 RAs within 6 months before the first instance of T2D and BM diagnosis. The control cohort included those with no GLP-1 RA use over the same period. We excluded those with potential contraindications to GLP-1 RA use and those with lower socioeconomic status (eAppendix 2 in Supplement 1). One-to-one propensity score (PS) matching between 2 groups was performed using the greedy nearest-neighbor algorithm with a caliper width of 0.1 pooled SDs (Table). A standardized mean difference less than 0.10 indicated balanced covariates. The primary outcome was all-cause mortality up to 3 years from the study index date—namely, the first recorded date of BM. We also conducted subgroup analyses based on primary cancer sites, GLP-1 RA types, and comparison with other antidiabetic agents. We also set seizure events and pneumonia as falsification end points to examine study findings’ vulnerability to unmeasured confounders. Cox proportional hazards models were used to estimate hazard ratios (HRs) and 95% CIs for the outcome. Statistical significance was defined as 2-tailed *P* < .05. All analyses were performed within the TriNetX Analytic Platform.

**Table.** Baseline Demographic and Clinical Characteristics Before and After Propensity Score Matching^a^

Characteristic	Before propensity score matching			After propensity score matching		
	Patients, No. (%)		*P* value	Patients, No. (%)		SMD
	GLP-1 RA (n = 866)	No GLP-1 RA (n = 11 103)		GLP-1 RA (n = 850)	No GLP-1 RA (n = 850)	
Age at index, mean (SD), y	64.3 (10.3)	67.7 (10.5)	<.001	64.5 (10.2)	65.0 (10.4)	0.050
Sex						
Male	393 (45.4)	5594 (50.4)	.005	387 (45.5)	377 (44.4)	0.024
Female	471 (54.4)	5505 (49.6)	.006	461 (54.2)	470 (55.3)	0.021
Race						
Asian	47 (5.4)	731 (6.6)	.18	46 (5.4)	43 (5.1)	0.016
Black or African American	120 (13.9)	1572 (14.2)	.81	117 (13.8)	119 (14.0)	0.007
White	622 (71.8)	7319 (65.9)	<.001	610 (71.8)	625 (73.5)	0.040
Other^b^	32 (3.7)	358 (3.2)	.45	32 (3.8)	32 (3.8)	<0.001
Unknown	34 (3.9)	969 (8.7)	<.001	34 (4.0)	25 (2.9)	0.058
Ethnicity						
Hispanic or Latino	54 (6.2)	608 (5.5)	.35	52 (6.1)	54 (6.4)	0.010
Not Hispanic or Latino	658 (76.0)	7914 (71.3)	.003	645 (75.9)	648 (76.2)	0.008
Unknown	154 (17.8)	2581 (23.2)	<.001	153 (18.0)	148 (17.4)	0.015
Malignant entity						
Lung cancer	315 (36.4)	4440 (40.0)	.04	312 (36.7)	306 (36.0)	0.015
Breast cancer	145 (16.7)	1601 (14.4)	.06	140 (16.5)	151 (17.8)	0.034
Melanoma	81 (9.4)	803 (7.2)	.02	80 (9.4)	71 (8.4)	0.037
Colon cancer	27 (3.1)	389 (3.5)	.55	27 (3.2)	22 (2.6)	0.035
Rectal cancer	12 (1.4)	149 (1.3)	.91	12 (1.4)	13 (1.5)	0.010
Kidney cancer	66 (7.6)	617 (5.6)	.01	63 (7.4)	56 (6.6)	0.032
Malignant neoplasm of larynx	10 (1.2)	51 (0.5)	.006	10 (1.2)	10 (1.2)	<0.001
Bone metastases	210 (24.2)	2679 (24.1)	.94	205 (24.1)	203 (23.9)	0.006
Comorbidities						
Essential hypertension	684 (79.0)	7776 (70.0)	<.001	672 (79.1)	690 (81.2)	0.053
Heart failure	153 (17.7)	1529 (13.8)	.001	150 (17.6)	156 (18.4)	0.018
Ischemic heart diseases	319 (36.8)	3298 (29.7)	<.001	309 (36.4)	316 (37.2)	0.017
Atrial fibrillation and flutter	109 (12.6)	1415 (12.7)	.89	106 (12.5)	102 (12.0)	0.014
Cerebrovascular diseases	257 (29.7)	2245 (20.2)	<.001	245 (28.8)	205 (24.1)	0.107
Morbid obesity	210 (24.2)	1103 (9.9)	<.001	199 (23.4)	204 (24.0)	0.014
Nicotine dependence	195 (22.5)	2587 (23.3)	.60	192 (22.6)	188 (22.1)	0.011
Alcohol-related disorders	30 (3.5)	458 (4.1)	.34	30 (3.5)	30 (3.5)	<0.001
Chronic kidney disease	234 (27.0)	2283 (20.6)	<.001	228 (26.8)	241 (28.4)	0.034
Other chronic obstructive pulmonary disease	192 (22.2)	2368 (21.3)	.56	190 (22.4)	197 (23.2)	0.020
Procedures						
SRS, multisource cobalt 60 based	24 (2.8)	158 (1.4)	.002	24 (2.8)	16 (1.9)	0.062
SRS, linear accelerator based	29 (3.3)	180 (1.6)	<.001	28 (3.3)	19 (2.2)	0.065
Stereotactic body radiation therapy	56 (6.5)	488 (4.4)	.005	53 (6.2)	36 (4.2)	0.090
Craniectomy or craniotomy procedures	68 (7.9)	340 (3.1)	<.001	62 (7.3)	51 (6.0)	0.052
Medications						
Antineoplastic therapy	418 (48.3)	4747 (42.8)	.002	411 (48.4)	405 (47.6)	0.014
Systemic corticosteroids	724 (83.6)	8033 (72.4)	<.001	711 (83.6)	693 (81.5)	0.055
Sodium-glucose cotransporter–2 inhibitors	248 (28.6)	727 (6.5)	<.001	236 (27.8)	229 (26.9)	0.018
Metformin	544 (62.8)	4352 (39.2)	<.001	530 (62.4)	543 (63.9)	0.032
Sulfonylureas	262 (30.3)	1995 (18.0)	<.001	258 (30.4)	257 (30.2)	0.003
Thiazolidinediones	83 (9.6)	382 (3.4)	<.001	79 (9.3)	87 (10.2)	0.032
Insulin	664 (76.7)	5393 (48.6)	<.001	648 (76.2)	659 (77.5)	0.031
Dipeptidyl peptidase–4 inhibitors	180 (20.8)	1188 (10.7)	<.001	177 (20.8)	182 (21.4)	0.014
Laboratory values, mean (SD)						
Hemoglobin A_1c_, %	7.9 (1.8)	7.0 (1.6)	<.001	7.8 (1.8)	7.5 (1.7)	0.219
Body mass index^c^	32.9 (7.4)	29.1 (6.8)	<.001	32.9 (7.4)	32.7 (6.9)	0.031
Estimated glomerular filtration rate, mL/min/1.73 m^2^	74.4 (33.1)	77.8 (33.7)	.007	74.4 (32.9)	75.7 (33.3)	0.039
Visit type						
Emergency department	456 (52.7)	5104 (46.0)	<.001	446 (52.5)	442 (52.0)	0.009
Inpatient encounter	518 (59.8)	5311 (47.8)	<.001	505 (59.4)	481 (56.6)	0.057
Ambulatory	778 (89.8)	9713 (87.5)	.04	763 (89.8)	776 (91.3)	0.052

Abbreviations: SMD, standardized mean difference; SRS, stereotactic radiosurgery.

SI conversion factor: To convert hemoglobin A_1c_ to proportion of total hemoglobin, multiply by 0.01.

^a^ Propensity score matching was performed on the following variables: age at index, sex, race, ethnicity, cancer types, hydrocephalus, morbid obesity, smoking, alcohol use, chronic kidney disease, other chronic obstructive pulmonary disease, heart failure, hypertension, atrial fibrillation or flutter, cerebrovascular diseases, ischemic heart diseases, radiation therapy, craniectomy or craniotomy, antineoplastic agents, sodium-glucose cotransporter–2 inhibitors, metformin, sulfonylureas, thiazolidinediones, insulin, dipeptidyl peptidase–4 inhibitors, hemoglobin A_1c_, body mass index, and estimated glomerular filtration rate.

^b^ Other race includes American Indian or Alaska Native, Native Hawaiian or Other Pacific Islander, and others.

^c^ Body mass index is calculated as weight in kilograms divided by height in meters squared.

## Results

Among a total of 19 234 patients with cancer, BM, and T2D, 866 GLP-1 RA users (semaglutide, 369 patients; dulaglutide, 379 patients; liraglutide, 147 patients; and tirzepatide, 95 patients), and 11 103 nonusers met eligibility criteria. After PS matching, 850 GLP-1 RA users were matched to nonusers, with well-balanced baseline characteristics (Table).

GLP-1 RA use was associated with significantly lower all-cause mortality (HR, 0.63; 95% CI, 0.54-0.72; *P* < .001) (Figure). The observed mortality benefit remains consistent across major cancer types and GLP-1 RA types, except for liraglutide, and in comparison with other antidiabetic therapy (Figure). Falsification end points were comparable between the 2 groups (Figure).

**Figure.**
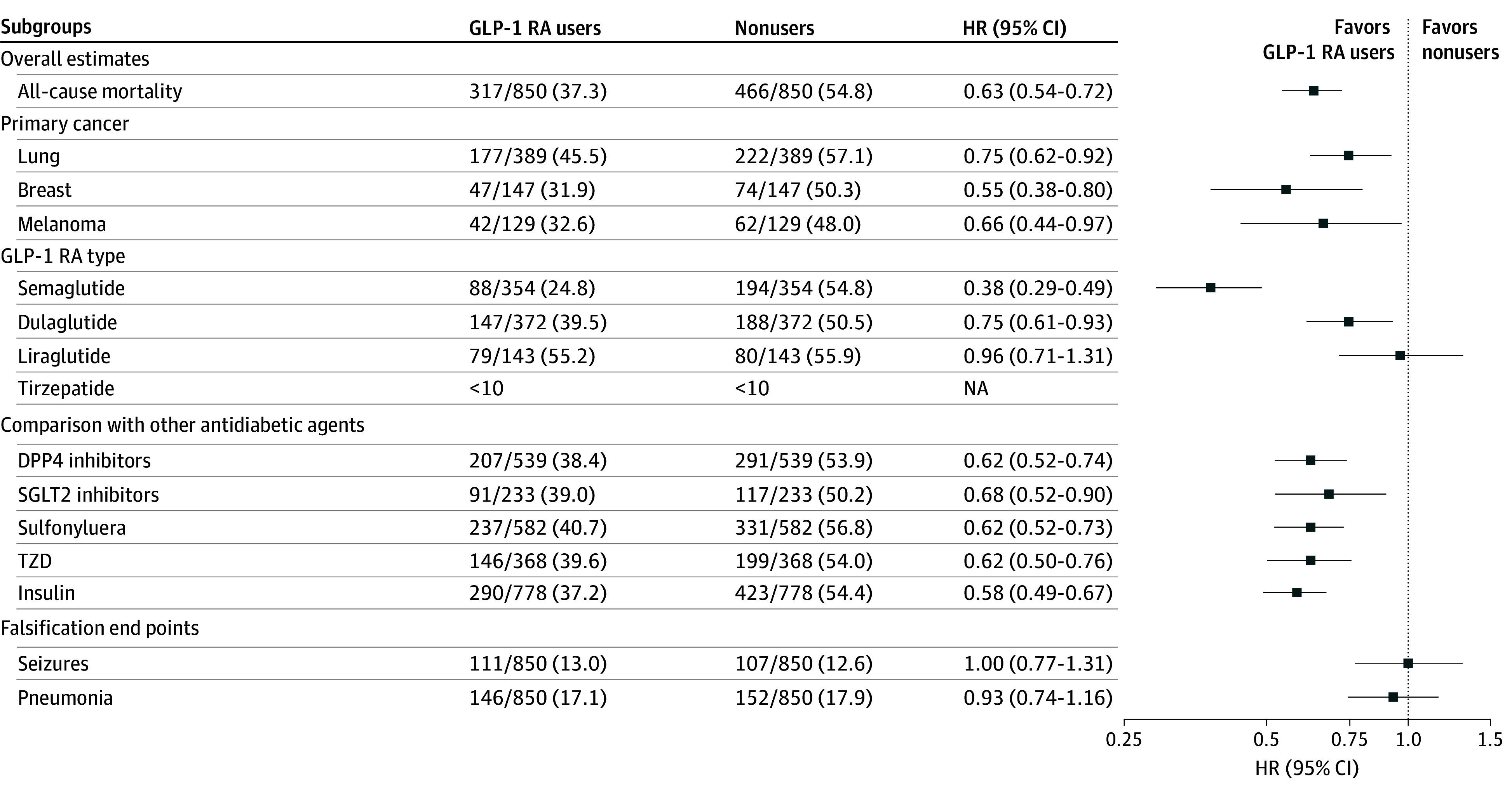
Forest Plot of Study Findings With Subgroup Analyses and Falsification End Points DPP4 indicates dipeptidyl peptidase–4; GLP-1 RA, glucagon-like peptide–1 receptor agonist; HR, hazard ratio; NA, not applicable; SGLT2, sodium-glucose cotransporter–2; TZD, thiazolidinedione.

## Discussion

The findings of this cohort study suggest that GLP-1 RA use was associated with significant reduction in all-cause mortality among patients with cancer with BM and T2D, with generally consistent association across subgroups. These results build upon existing evidence that GLP-1 receptor activation modulates pathways relevant to neuro-oncologic health, including attenuation of neuroinflammation, preservation of blood–brain barrier integrity, and reduction of oxidative stress and mitochondrial dysfunction.^5,6^

Our study has several limitations. First, the retrospective design limits causal inference despite PS matching and comparable falsification end points. Second, the lack of individual-level data precluded dose-response analyses, detailed assessment of concomitant systemic and radiation therapies, and evaluation of cancer-specific mortality. Third, the restriction of 1:1 PS matching within TriNetX excluded substantial nonusers, limiting the generalizability of our findings to patients with sufficient covariate overlap. Fourth, participating health care organizations within TriNetX are mostly academic hospitals, also limiting the generalizability of our findings to nonacademic setting. GLP-1 RA was associated with significant reduction in all-cause mortality among patients with cancer with BM and T2D, warranting future prospective studies to further elucidate the effects of GLP-1 RA in cancer populations.

## Notes

Supplement 1. eAppendix 1. Data Source eReferences eAppendix 2. Diseases, Medications, and Outcome Definition

Supplement 2. Data Sharing Statement
