# Supplementary material for: GLP-1 Receptor Agonist Use and Survival Among Patients With Type 2 Diabetes and Brain Metastases
Source: JAMA Netw Open. 2026 Mar 11;9(3):e261311. doi: 10.1001/jamanetworkopen.2026.1311 (PMC12980253; doi:10.1001/jamanetworkopen.2026.1311)
Supplement: Supplement 1. — eAppendix 1. Data Source eReferences eAppendix 2. Diseases, Medications, and Outcome Definition [file jamanetwopen-e261311-s001.pdf]

Supplemental Online Content

Chi K-Y, Chang Y, Song J, Chen C-M, Perng P-S, Lin H-M. GLP-1 receptor agonist use and survival among patients with type 2 diabetes and brain metastases. *JAMA Netw Open*. 2026;9(3):e261311. doi:10.1001/jamanetworkopen.2026.1311

eAppendix 1. Data Source
eReferences
eAppendix 2. Diseases, Medications, and Outcome Definition

This supplemental material has been provided by the authors to give readers additional information about their work.

© 2026 Chi K-Y et al. *JAMA Network Open*.

eAppendix 1. Data Source

This is a retrospective cohort study utilizing the TriNetX US Collaborative Network database which provides access to approximately 120 million of patients' electronic medical records from 70 healthcare organizations (HCOs) in the United States across over 50 states, including 39% South, 22% Northeast, 16% Midwest, 13% West, and 10% Unspecified).[1] Data in TriNetX is primarily derived from HCOs' electronic health records (EHRs), supplemented by connecting to additional sources, including billable codes from closed claims, the Social Security Administration Master Death File, private obituaries, and private claims. TriNetX uses a standardized data mapping process to convert healthcare data from aforementioned sources into uniform terminologies, including International Classification of Diseases, Tenth Revision, Clinical Modification (ICD-10-CM) codes, International Classification of Diseases, Tenth Revision, Procedure Coding System (ICD-10-PCS), RxNorm, Anatomical Therapeutic Chemical (ATC) Classification, and Current Procedural Terminology (CPT) code. For mortality data, the term "Deceased" was used to capture death. Mortality data in TriNetX is primarily derived from HCO death records, which are sourced from EHRs. However, recognizing the inherent limitations of HCO data—where mortality is primarily recorded for patients who die during hospitalization—TriNetX supplements this by connecting to additional sources, including billable codes from closed claims, the Social Security Administration Master Death File, private obituaries, and private claims. Mortality data directly from HCOs uses the ICD-10-CM code R99 to map the "Deceased" term. For patients with additional mortality data from the aforementioned external sources, these are also mapped to the "Deceased" term to improve data completeness. Sex, race, and ethnicity were classified based on documentation in EHRs, typically derived from patient self-report at the time of clinical encounter. Missing data in sex were categorized as "unknown sex," and missing race or ethnicity were classified as "unknown race or ethnicity." Researchers build up cohort queries on the TriNetX online portal using these standardized terminologies.

Moreover, it's particularly useful to harness the built-in statistical software which allows researchers to efficiently obtain outcomes interested in. The construct of study query and the study outcomes were primarily based on these codes. Subsequently, designed cohorts are sent to the Advanced Analytics Platform with built-in statistical function to perform analyses of interest. TriNetX's data was validated for data completeness[2] and has been successfully used to conduct numerous comparative effectiveness research,[3-5] including those in the context of cardiovascular disease research.[6,7] TriNetX is compliant with the Health Insurance Portability & Accountability Act (HIPAA) as the platform only reveals de-identified aggregate-level patient information. Therefore, this study is exempt from the institutional review board.

© 2026 Chi K-Y et al. *JAMA Network Open*.

eReferences:

1. Palchuk MB, London JW, Perez-Rey D, et al. A global federated real-world data and analytics platform for research. *JAMIA Open.* 2023;6(2):ooad035.
2. Evans L, London JW, Palchuk MB. Assessing real-world medication data completeness. *J Biomed Inform.* 2021;119:103847.
3. Wan G, Chen W, Khattab S, et al. Multi-organ immune-related adverse events from immune checkpoint inhibitors and their downstream implications: a retrospective multicohort study. *Lancet Oncol.* 2024;25(8):1053-1069.
4. Pan HC, Chen JY, Chen HY, et al. GLP-1 receptor agonists' impact on cardio-renal outcomes and mortality in T2D with acute kidney disease. *Nat Commun.* 2024;15(1):5912.
5. Wang L, Davis PB, Kaelber DC, Volkow ND, Xu R. Comparison of mRNA-1273 and BNT162b2 Vaccines on Breakthrough SARS-CoV-2 Infections, Hospitalizations, and Death During the Delta-Predominant Period. *Jama.* 2022;327(7):678-680.
6. Avula V, Sharma G, Kosiborod MN, et al. SGLT2 Inhibitor Use and Risk of Clinical Events in Patients With Cancer Therapy-Related Cardiac Dysfunction. *JACC Heart Fail.* 2024;12(1):67-78.
7. Satti DI, Karius A, Chan JSK, et al. Effects of Glucagon-Like Peptide-1 Receptor Agonists on Atrial Fibrillation Recurrence After Catheter Ablation. *JACC Clin Electrophysiol.* 2024;10(8):1848-1855.

© 2026 Chi K-Y et al. *JAMA Network Open*.

eAppendix 2. Diseases, Medications, and Outcome Definition

**Diseases or Procedures**		
Type 2 diabetes mellitus	UMLS:ICD10CM:E11	
Secondary malignant neoplasm of brain	UMLS:ICD10CM:C79.31	
Multiple endocrine neoplasia syndromes	UMLS:ICD10CM:E31.2	
Alcohol-induced chronic pancreatitis	UMLS:ICD10CM:K86.0	
Other chronic pancreatitis	UMLS:ICD10CM:K86.1	
Malignant neoplasm of thyroid gland	UMLS:ICD10CM:C73	
Dialysis Services and Procedures	UMLS:CPT:1012740	
End stage renal disease	UMLS:ICD10CM:N18.6	
Hemodialysis Procedures	UMLS:CPT:1012752	
Dependence on renal dialysis	UMLS:ICD10CM:Z99.2	
Persons with potential health hazards related to socioeconomic and psychosocial circumstances	UMLS:ICD10CM:Z55-Z65	
**Medications**		
**Glucagon-like peptide-1 analogues**	NLM:ATC:A10BJ	
**tirzepatide**	NLM:RXNORM:2601723	
**Outcomes**		
All-cause mortality	Deceased	Deceased
Falsification endpoints–1	UMLS:ICD10CM:G40	Epilepsy and recurrent seizures
Falsification endpoints–2	UMLS:ICD10CM:J18	Pneumonia, unspecified organism

© 2026 Chi K-Y et al. *JAMA Network Open*.
