# Supplementary material for: GLP-1 Receptor Agonist Use and Survival Among Patients With Type 2 Diabetes and Brain Metastases
Source: JAMA Netw Open. 2026 Mar 11;9(3):e261311. doi: 10.1001/jamanetworkopen.2026.1311 (PMC12980253; doi:10.1001/jamanetworkopen.2026.1311)
Supplement: Supplement 2. — Data Sharing Statement [file jamanetwopen-e261311-s002.pdf]

Data Sharing Statement

Chi. GLP-1 Receptor Agonist Use and Survival Among Patients With Type 2 Diabetes and Brain Metastases. *JAMA Netw Open*. Published March 11, 2026.
doi:10.1001/jamanetworkopen.2026.1311

Data
Data available: No
